# Characters of homogentisate oxygenase gene mutation and high clonality of the natural pigment-producing *Vibrio cholerae *strains

**DOI:** 10.1186/1471-2180-11-109

**Published:** 2011-05-18

**Authors:** Ruibai Wang, Hengliang Wang, Haijian Zhou, Yuelan Wang, Junjie Yue, Baowei Diao, Biao Kan

**Affiliations:** 1 State Key Laboratory for Infectious Disease Prevention and Control, National Institute for Communicable Disease Control and Prevention, Chinese Center for Disease Control and Prevention, Beijing 102206, PR China; 2 Beijing Institute of Biotechnology, State Key Laboratory of Pathogen and Biosecurity, Beijing 100071, PR China

**Keywords:** *Vibrio cholerae*, pigment, homogentisate oxygenase, clonality

## Abstract

**Background:**

Some microorganisms can produce pigments such as melanin, which has been associated with virulence in the host and with a survival advantage in the environment. In *Vibrio cholerae*, studies have shown that pigment-producing mutants are more virulent than the parental strain in terms of increased UV resistance, production of major virulence factors, and colonization. To date, almost all of the pigmented *V. cholerae *strains investigated have been induced by chemicals, culture stress, or transposon mutagenesis. However, during our cholera surveillance, some nontoxigenic serogroup O139 strains and one toxigenic O1 strain, which can produce pigment steadily under the commonly used experimental growth conditions, were obtained in different years and from different areas. The genes VC1344 to VC1347, which correspond to the El Tor strain N16961 genome and which comprise an operon in the tyrosine catabolic pathway, have been confirmed to be associated with a pigmented phenotype. In the present study, we investigated the mechanism of pigment production in these strains.

**Results:**

Sequencing of the VC1344, VC1345, VC1346, and VC1347 genes in these pigmented strains suggested that a deletion mutation in the homogentisate oxygenase gene (VC1345) may be associated with the pigmented phenotype, and gene complementation confirmed the role of this gene in pigment production. An identical 15-bp deletion was found in the VC1345 gene of all six O139 pigment-producing strains examined, and a 10-bp deletion was found in the VC1345 gene of the O1 strain. Strict sequence conservation in the VC1344 gene but higher variance in the other three genes of this operon were observed, indicating the different stress response functions of these genes in environmental adaption and selection. On the basis of pulsed-field gel electrophoresis typing, the pigment-producing O139 strains showed high clonality, even though they were isolated in different years and from different regions. Additionally all these O139 strains belong to the rb4 ribotype, which contains the O139 strains isolated from diarrheal patients, although these strains are cholera toxin negative.

**Conclusion:**

Dysfunction of homogentisate oxygenase (VC1345) causes homogentisate accumulation and pigment formation in naturally pigmented strains of *V. cholerae*. The high clonality of these strains may correlate to an environmental survival advantage in the *V. cholerae *community due to their pigment production, and may imply a potential protective function of melanin in environmental survival of such strains.

## 1. Background

*Vibrio cholerae *is the etiological agent of the severe diarrheal disease cholera. It has caused seven pandemics since 1817. The seventh pandemic, which began in 1961, was triggered by biotype El Tor, serogroup O1. In 1991, a new serogroup, O139, appeared, challenging the common belief that only strains of the O1 serogroup could cause epidemics [1,2]. Epidemics of cholera caused by O1 and O139 *V. cholerae *are still a major public health problem in most developing countries. In addition to the known cholera toxin and colonization factors of *V. cholerae*, many other factors contribute to the pathogenicity of this organism, including hemolysin, RTX toxin, and adaptive response systems [3-6]. The environmental survival ability of this microorganism, which has two life cycles, is very important. Climate and environmental changes, including temperature of the aquatic environment [7] and seasonal algal blooms [8], have been confirmed to be related to the persistence and outbreak of cholera in human populations [9].

In addition to the well-studied virulence factors, melanin has also been linked with pathogenicity and virulence in a variety of pathogenic microbes, including *Cryptococcus neoformans, Azotobacter chroococcum*, group B *Streptococcus*, and *Burkholderia cepacia *[10-12], and its catabolic pathway has became an important herbicide target in plants [13,14]. Melanin is the most widely distributed protective pigment in the biosphere and its production is thought to be of great significance [15-17]. Considerable interest has been shown in melanin, apart from its association with severe human diseases. Melanin is believed to contribute to microbial virulence and provides a survival advantage by increasing a pathogen's tolerance to enzymatic degradation, radiation (UV, solar, or gamma), heavy metals, and adverse temperatures (heat and cold); by reducing a pathogen's susceptibility to killing through host antimicrobial mechanisms; and by interfering with the host immune response to infection [10-12]. For *V. cholerae*, it has been reported that mutants induced by chemical reagents or natural isolates subjected to stress, particularly hyperosmotic shock and elevated temperature, can produce brown pigment [18-22]. Melanogenesis also has a specific function with respect to the survival of *V. cholerae *in its natural habitats [20]. A further study has shown that melanin pigment formation can enhance the viability of *V. cholerae *strains in terms of UV resistance, the production of major virulent factors, and colonization, and that mutants of *V. cholerae *that produce large amounts of melanin are more virulent than their non-melanogenic parental strain [23].

In the tyrosine catabolic pathway, melanin pigment is produced [23,24] from homogentisate, which is the main *p*-diphenolic intermediate of normal L-tyrosine catabolism. After its formation through this pathway, the aromatic ring undergoes an oxidative cleavage to yield maleylacetoacetate, which is *cis*:*trans *isomerized to fumarylacetoacetate, and this compound is finally split into fumarate and acetoacetate. The enzymes involved in this pathway are, successively, *p*-hydroxyphenylpyruvate dioxygenase (*p*HPD), homogentisate oxygenase (HGO), maleylacetoacetate isomerase (MAI), and fumarylacetoacetate hydrolase (FAH). It is known that disruption of the balance between the first two enzymes in this sequence can cause homogentisate accumulation, and leads to its spontaneous oxidation and the production of the so-called pyomelanins [15]. *V. cholerae *has been proposed to be a useful prokaryotic model of alterations in L-tyrosine catabolism and has been used to study the molecular basis of diseases related to L-tyrosine catabolism [15]. However, to date, all the research on melanogenesis in *V. cholerae *has been based on chemically induced mutants or mutants generated using transposons.

During our cholera surveillance, some O139 and O1 strains that produced soluble brown pigments were isolated from environmental water samples and patients. Unusually, these strains can produce pigment under the normally used experimental growth conditions [Luria-Bertani (LB) nutrient agar or broth without temperature limitation]. Using transposon mutagenesis, we determined that the *p*-hydroxyphenylpyruvate dioxygenase (HPD; VC1344 in the N16961 genome) in the tyrosine catabolic pathway was responsible for the pigment production in these strains [24]. Further, the three genes in a cluster downstream of VC1344 were found to correspond to the other three enzymes involved in tyrosine catabolism [23,24]. In this study, we analyzed the sequence variance of the four genes involved in tyrosine catabolism and the functions of the mutant genes to determine the possible mechanism of pigment production in these isolates. We also found a close relationship of clonality among these strains, even though they were isolated in different years and from different areas. The potentiality of clone selection and pathogenicity of such strains should be considered.

## 2. Methods

### 2.1 *Strains*

In this study, 22 *V. cholerae *O1 and O139 toxigenic and nontoxigenic strains were used (Table 1). Among these isolates, 95-4, 98-200, JX2006135, JX2006136, JX2006175, GD200101012, and 3182 are pigment-producing strains. These strains were isolated in different years and from different provinces of China. The El Tor strain 3182 was isolated from patients and the other six O139 strains were isolated from environmental water. In addition to the reference strains, including N16961, 569B, and MO45, the controls included other non-pigment-producing strains that were isolated in the same province or at the same time as the pigmented strains. Strains were cultured in LB liquid medium shaking at 37°C or on LB agar plates (1% tryptone, 0.5% yeast extract, 0.5% NaCl, and 1.5% agar).

**Table 1:** Strains used in this study and relative characters

	Strain	Serogroup/Biotype	Location (province)	Year of isolation	Source	Cholerae toxin gene	Pigment
1	N16961	O1 El Tor	Bangladesh	1971	Patient	+	-
2	569B	O1 classical	Calcutta, India	1948	Patient	+	-
3	7743	O1 El Tor	Guangdong	1977	Water	-	-
4	3182	O1 El Tor	Guangdong	1994	Patient	+	+
5	JS32	O1 El Tor	Jiangsu	1990	Water	-	-
6	WJ-2	O1 El Tor	Jiangsu	1980	Patient	+	-
7	98-200	O139	Guangdong	1998	Water	-	+
8	95-4	O139	Guangdong	1995	Water	-	+
9	JX2006136	O139	Jiangxi	2006	Water	-	+
10	JX2006175	O139	Jiangxi	2006	Water	-	+
11	GD200101012	O139	Guangdong	2001	Water	-	+
12	JX2006135	O139	Jiangxi	2006	Water	-	+
13	MO45	O139	Madras, India	1992	Patient	+	-
14	98-514	O139	Anhui	1998	Water	-	-
15	96-84	O139	Anhui	1996	Water	-	-
16	94001	O139	Xinjiang	1994	Water	-	-
17	GD2006080	O139	Guangdong	2006	Water	-	-
18	JX2006101	O139	Jiangxi	2006	Water	-	-
19	JX2006102	O139	Jiangxi	2006	Water	-	-
20	JX2006177	O139	Jiangxi	2006	Water	-	-
21	JX2006127	O139	Jiangxi	2007	Water	-	-
22	JX2006129	O139	Jiangxi	2008	Water	-	-

* Provinces are presented when the strain is from China.

^# ^Pigment which can be observed in the culture condition of LB medium and 37°C.

### 2.2 PCR and sequencing

Four genes of VC1344, VC1345, VC1345, and VC1347 (corresponding to the N16961 genome) were amplified using the primer pairs listed in Table 2 (S-1344, S-1345, S-1346 and S-1347 respectively). The PCR products were purified and sequenced. Sequence alignments and comparisons were performed using the CLUSTAL X program (version 2.0).

**Table 2:** Primers used in this study

Primer pairs	Primer sequences	
S-1344	U	5' AAG GCA AGG GTT TTT GTG 3'
	L	5' TGT CGG TGC ATG TTG ATG 3'
S-1345	U	5' GCG CAA AGG TAA TCA AGG 3'
	L	5' GTT ATC CAA CGC CTG CTG 3'
S-1346	U	5' GCA GCA GGT GGA AAA TCG 3'
	L	5' ATT GAG GGC AAT ACG CAC 3'
S-1347	U	5' TTT TTG GTG CGA TTG AGC 3'
	L	5' TGC CGA TGA AGA ATC TGC 3'
RT-1344	U	5' TTT GTG GAT CGT TAT GGC 3'
	L	5' AAT GCC ATC TTT CAT CGG 3'
RT-1344-45	U	5' TGC ACC GAT GAA AGA TGG 3'
	L	5' CAC CCG CAC TTT CAC TTC 3'
RT-1345	U	5' GAA GTG AAA GTG CGG GTG 3
	L	5' TTG GAA CGC TTT CGG ATG 3'
RT-1345-46	U	5' CAT CCG AAA GCG TTC CAA 3'
	L	5' AAA TCT CGG CTC ACC ACC 3'
RT-1346	U	5' GGT GGT GAG CCG AGA TTT 3'
	L	5' GCG ACA GGT GAA AAA GCC 3'
RT-1346-47	U	5' ACA CGA GCA CTG TGT GCG 3
	L	5' GGC GCG TGA CTC GTA AAC 3'
RT-1347	U	5' AGC ATC ATG CCG AGT TTC 3'
	L	5' ATA TTC CCC TGC CGT ATG 3'
1345:1U	U	5' CAT GCC ATG GAT GCA TAA ATG GAT C 3'
1345:525L	L	5' GAT CGA AGG CAC GTC CAA CAC GGC AGG ATC AAA CAC CGC GTG ATT G 3'
1345:555U	U	5' GGA CGT GCC TTC GAT C 3'
1345:1122L	L	5' CAT GCC ATG GCT ACT CCT TTT TAC TC 3'
16S	U	5' AGA GTT TGA TCA TGG CTC AG 3'
	L	5' AAG GAG GTG ATC CAA CCG CA 3'

Reverse transcription PCR was used to detect if these four genes were transcribed together. Total RNA of strains N16961 and 95-4 was extracted using an RNeasy Mini Kit (Qiagen), transcribed to cDNA and used as templates. Four pairs of primers designed within of the ORF of each gene, RT-1344, RT-1345, RT-1346 and RT-1347 (Table 2), and three pairs of primers spanning the intervals between these four genes, RT-1344-45, RT-1345-46, and RT-1346-47 (Table 2), were used in the amplification. The total mRNA without reverse transcription were used as negative control,

### 2.3 Filling in of the 15-bp gap in the VC1345 gene

**Figure 1:**
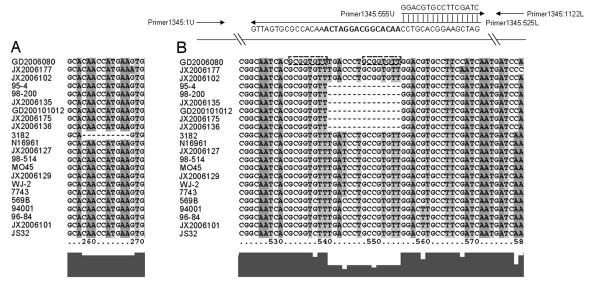
**The aligning maps of the sequences of VC1345 gene and the schematic diagram of the primers used in the function analysis of the 15bp gap of the VC1345 gene of the O139 pigment producing *V. cholerae *strains**. A. Mutation of the strain 3182 compared to other strains. B. Mutation of the O139 pigment producing strains. Two dashed boxes up the VC1345 gene sequence showed the short direct repeat at the deletion breakpoint.

Two pairs of primers were used to amplify the upstream and downstream fragment of the 15-bp gap in the VC1345 gene of pigment-producing strain 95-4. The primers were as follows: 1345:1U, 1345:525L, 1345:555U and 1345:1122L (Figure 1 and Table 2). The 5' end of primer 1345:525L overlapped with 1345:555U and contained the 15-bp gap sequence. Apart from the 15-bp gap sequence, the PCR product has the same sequence as the wild-type VC1345 gene of 95-4. The PCR fragment was then cloned into the *Nco*I enzyme site of the expression vector pET15b (No. 69661-3; Novagen, Germany) and transformed into wild-type strain 95-4. The original VC1345 gene of 95-4 was also amplified and cloned into pET15b, then transformed into 95-4 as a control.

### 2.4 Ribotyping

Chromosomal DNAs of the test strains were extracted and digested with the enzyme *Bgl*I. DNA fragments were separated and transferred to nylon membranes. The membranes were prehybridized at 42°C for 2 h in hybridization solution without probe (2× SSC, 1% block reagent, 0.1% *N*-lauryl sarcosine, 0.02% SDS, and 50% formamide) and then hybridized with the freshly denatured labeled gene probes at 42°C for 12 h. Hybridized membranes were washed twice in 2× SSC-0.1% SDS for 5 min at room temperature, followed by two washes in 0.1× SSC-0.1% SDS for 15 min at 68°C. The probe used in this typing was the PCR product of the conserved 16S rRNA gene of *Escherichia coli*, which was amplified by primers 5'-TTT AAT GAC CAG CAC AGT-3' and 5'-TCT GCC AGT GTT ACA ACC-3', and was labeled using a random primer DIG DNA Labeling and Detection Kit (Roche Molecular Biochemicals, Indianapolis, IN). Detection was based on digoxigenin-anti digoxigenin ELISA, according to the manufacturer's instructions.

### 2.5 Pulsed-field gel electrophoresis (PFGE)

The PFGE protocol used was based on the PulseNet 1-day standardized PFGE protocol for *V. cholerae *[25]. The cell suspension in a polystyrene tube (Falcon; 12 by 75 mm) was adjusted to an optical density of 4.0-4.2 using bioMerieux DENSIMAT; *V. cholerae *slices were digested with 20 U per slice *Not*I (New England Biolabs) for 4 h at 37°C. Electrophoresis was performed using a CHEF-DRIII system (Bio-Rad Laboratories). Images were captured using a Gel Doc 2000 system (Bio-Rad) and converted to TIFF files for computer analysis. The BioNumerics software package (version 4.0; Applied Maths, Inc.) was used to analyze the PFGE patterns. Fragments smaller than 20.5 kbp were not taken into account. Similarity analysis was performed by calculating Dice coefficients (S_D_), with customized tolerance for each EP. S_D _was calculated as follows:

where n*_xy _*is the number of bands common to isolates *x *and *y*, n*_x _*is the total number of bands for isolate *x*, and n*_y _*is the total number of bands for isolate *y*. The tolerance was determined according to the value when all the patterns obtained with the same EP were defined to be indistinguishable. Clustering was created using the unweighted-pair group method using average linkages (UPGMA).

### 2.6 Nucleotide sequence accession numbers

The GenBank accession numbers for the nucleotide sequences determined in this study are as follows: VC1344, GU930289 to GU930308; VC1345, GU942498 to GU942519; VC1346, GU942520 to GU942541; and VC1347, GU942542 to GU942562.

## 3. Results

### 3.1 Sequence variation in the VC1344 to VC1347 gene cluster

**Figure 2:**
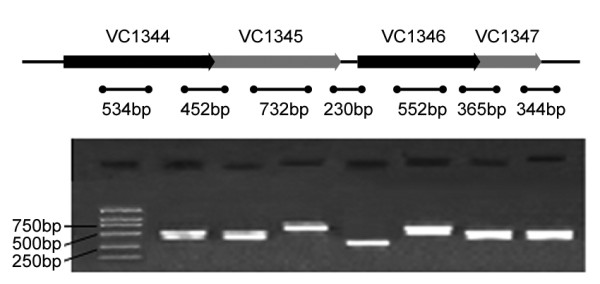
**Transcription analysis of VC1344 to VC1347**. The short lines with two dots at both ends indicate the location of primer pairs (sequences are listed in Table 2) used in reverse transcription PCR and the expected amplicons. The electrophoresis gel showed the reverse transcription PCR results, the lanes were arranged with the order of the upper amplicons.

In most cases, the chromosomal location of the HPD gene is next to other genes with no functional relationships; however, in *V. cholerae*, this gene is linked to the other genes involved in tyrosine metabolism, which were annotated as products of VC1344 to VC1347 [26]. Using the total mRNA of N16961 and 95-4 cultures as templates, reverse transcription PCR showed that all the three intervals of these four genes were amplified (Figure 2), whereas the total mRNA without reverse transcription (negative control) were negative, which indicated that VC1344 to VC1347 were transcribed as a single primary RNA and thereby constituted an operon in *V. cholerae*.

**Figure 3:**
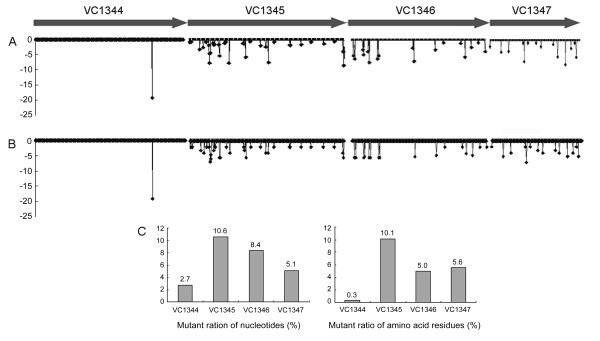
**Mutation frequency of the protein sequences of VC1344, VC1345, VC1346 and VC1347**. Software al2co is used in this analysis. The conservation calculation method is Sum-of-pairs measure and gap fraction to suppress calculation is 0.50. A. The frequency obtained in the comparison of all the tested strains. B. The frequency of the non-pigment producing strains. C. Histogram of the mutant ratios of the nucleotides and amino acid residues of the four genes.

The four genes VC1344 to VC1347 of the 22 strains listed in Table 1 were sequenced. Each gene and the predicted proteins with the number of the mutant sites, and the frequencies of mutation are shown in Figure 3. These results show that the four genes within a single operon exhibit different levels of variation. VC1344 is the most conserved and VC1345 has the highest variance, with mutation rates of 2.7% and 10.6% at the nucleotide level, respectively. This difference in mutation rate was also evident in the non-pigment-producing strains (Figure 3B). Although the VC1344 gene has 30 mutant sites in its nucleic acid sequence, only one mutant residue was found in its amino acid sequence at position 293, which is either Ala or Val. This one residue substitution does not cause polar or acid-alkaline change. On the basis of this amino acid residue difference, the test strains can be divided into two groups. Strains in the Val293 group include O1 (classical and El Tor) and O139 strains, whereas all of the strains in the Ala293 group belong to serogroup O139, including all six of the O139 pigment-producing strains. Because non-pigment-producing strains are also placed in this group, it can be presumed that this genotype is unrelated to pigment production. Moreover, none of the mutant sites found in the VC1346 and VC1347 genes were consistently present in genomes of the pigment-producing strains.

**Figure 4:**
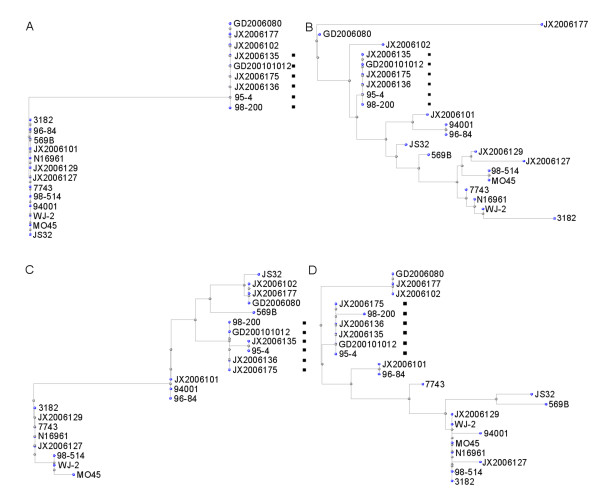
**The cluster analysis of the protein sequences of the four tyrosine catabolic genes, VC1344 (A), VC1345 (B), VC1346 (C) and VC1347 (D)**. Strains marked with black square are pigment producing strains.

Among the pigment-producing strains, sequences of the four genes in the O1 strain 3182 were the same as those in N16961; the exception being VC1345, in which a 10-bp sequence was missing between nucleotides 258 and 267. This caused a frameshift mutation and complete change in its protein sequence (Figure 1). Among the six O139 pigment-producing strains, the sequences of the four genes were almost identical, with the exception of four nucleotide differences: in the VC1346 gene, C591 in JX2006135, and A863 in JX2006135 and 95-4; and in the VC1347 gene, A1 in 98-200. Because of the high similarity identified in the cluster analysis of these four genes, all of the six pigment-producing strains could be grouped into one cluster, and, with the exception of the VC1344 gene, none of the non-pigment-producing strains was included in the clusters of the pigment-producing strains (Figure 4). In VC1345, a 15-bp fragment deletion, from nucleotide 539 to 554, was found in all six of the O139 pigment-producing strains, suggesting that this deletion mutation may be correlated with their pigment phenotype. In the borders of the deletion region, a short direct repeat (GCGGTGTT) was found (Figure 1).

### 3.2 Functional complementation of the VC1345 gene of strain 95-4

Using overlap PCR (Figure 1), we obtained the fragment which contain the complementary 15 nt which is absent in the wild pigment production strain 95-4, corresponding to the filling in the 15-bp gap in the VC1345 and retained the remainder of the gene sequence as in the pigment production wild-type. We then cloned this fragment containing backbone of the wild-type VC1345 gene of strain 95-4 and the 15 nt filling, into the expression vector pET15b and this recombinant plasmid was transformed into the wild-type 95-4 strain. This gene was expressed with induction of IPTG. After *trans*-complementation, strain 95-4 with the plasmid carrying the 15 nt filling of VC1345 gene no longer produced pigment, whereas the control strain 95-4 containing its own VC1345 gene cloned in pET15b showed no change in its pigment producing ability. This therefore showed that providing HGO enzyme is sufficient to avoid the pigment production and filling in of the 15-bp gap is sufficient to recover VC1345 gene function.

### 3.3 Clonality of the pigment-producing strains

**Figure 5:**
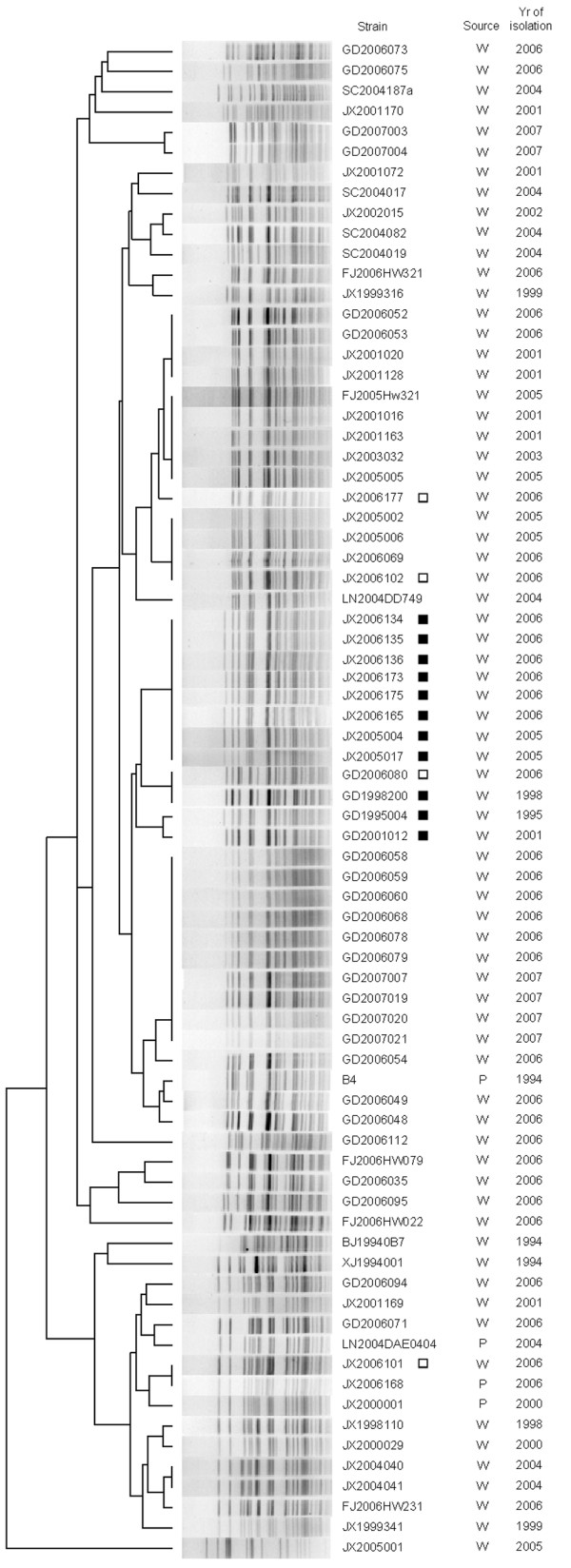
**The PFGE phylogenetic tree of the O139 pigment producing strains and other O139 non-toxigenic strains**. Strains marked with black square are pigment producing strains and white square are non-pigment producing strains which are included in the VC1344-VC1347 sequence analysis.

The pigment-producing strains, particularly those in serogroup O139, have the same mutation in the HPD gene; however, these strains were isolated in different years and from different areas. To explore the clonal relationships among these strains and the other strains, we used molecular typing methods to compare the strains at the genome level. In the PFGE analysis, the patterns of the six O139 pigment-producing strains were compared with the other nontoxigenic O139 strains in our *V. cholerae *PFGE database, which covers the O139 strains isolated in China from 1993 and the O1 strains isolated from 1961. The cluster analysis (Figure 5) showed that all of the 11 pigment-producing strains could be grouped together and separated from other non-pigment-producing strains, including some strains isolated in the same year and from the same province as the pigment-producing strains. Strain 3182 was not included in the PFGE analysis since it has an O1 serogroup.

Previously, we analyzed the ribotyping polymorphism of O139 isolates collected since O139 cholera appeared in China [27]. Here, we also determined the ribotypes of these pigment-producing strains. Hybridization showed that all of the O139 pigment-producing strains had the same ribotype, which was the same as the rb4 type identified in our previous study. The El Tor strain 3182 has a similar pattern to the toxigenic strain N16961.

## 4. Discussion

Many environmental microbes produce melanins, and melanin pigments are also an inherent phenotype of a broad range of eukaryotic microorganisms. The melanin in these strains may confer resistance to unfavorable environmental factors, host immunity, and even play a role in virulence expression. Therefore, melanin may confer a survival advantage on these natural pigment-producing *V. cholerae *strains in the estuary niche, and pathogenicity in the host.

Previously, *V. cholerae *strains with a pigmented phenotype were induced under stress or by chemical mutagenesis. In this study, we describe certain O139 and O1 isolates that can produce pigment under normal experimental growth conditions. Though the mutations in these O1 and O139 pigment-producing strains are different, both of them involve the dysfunction of HGO, the product of the VC1345 gene of *V. cholerae*. In our study, gene complementation of the mutant VC1345 confirmed the role of its dysfunction in pigment production. As a consequence, the disruption of the balance between the enzymes encoded by VC1344 and VC1345 causes homogentisate accumulation and spontaneous oxidation. The pigment production mechanism in these wild-type strains is same as in the chemically induced pigmented mutants [15,18].

With the exception of the 10-bp deletion in the VC1345 gene of the El Tor strain 3182, which appears to be a random mutation, it is interesting that the same 15-bp deletion in the VC1345 gene occurs in all the studied O139 pigment-producing strains, despite the fact that these strains were isolated over a period spanning the years 1995 to 2006. The deletion boundaries contain short direct repeats; therefore, it is possible that these commonly occurring recombinations gave rise to the mutant strains. It is not clear, however, how the 15-bp fragment affects the activity of the HGO enzyme. In the crystal structure of human HGO [28], the homologous amino acid residues encoded by this 15 bp form a small turn in the protein surface. Although it is not included in the predicted active sites or the 20 missense mutations that have been identified in the HGO from AKU patients [28], structural change in this mutant protein could be assumed.

The genes VC1344, VC1345, VC1346, and VC1347 comprise an operon, and the products of all four genes are predicted to be involved in tyrosine catabolism. The nucleotide and amino acid sequence variations in these genes are, however, inconsistent; VC1344 is highly conserved, although its nucleotide sequence varies among the different strains, only a single amino acid residue difference is present at the protein level, which suggests that it plays an important role in the tyrosine pathway, and is conserved despite undergoing different stress selections. In contrast, VC1345 is considerably more variable, and different deletion mutations result in dysfunction of its product. This suggests that the accumulation of homogentisate, and the subsequent melanin production instead of complete decomposition of the amino acid in the routine pathway, may have survival benefits for the mutants in certain specific environments, thus the mutations will be retained. Variation and even dysfunction of the VC1345 product may shift the metabolic production of tyrosine and produce strains that are adapted to surviving in rigorous environments.

It is also interesting that the molecular types of the O139 pigment strains are indistinguishable or quite similar, suggesting the high clonality of these strains, even though they were obtained over a span of at least 12 years and from different regions. They have the same mutation in the tyrosine metabolism pathway. Additionally, compared to the high variance of the VC1344 to VC1347 genes, the sequences in all the six O139 pigment-producing strains were highly consistent. These data suggest that the O139 pigment-producing strains originate from one distinctive clone. The wide distribution of such strains in the environment may suggest their survival advantage. The signature of the 15-bp deletion within the homogentisate 1,2-dioxygenase gene (VC1345) in the O139 pigmented strains, or the mutation of VC1345 in the melanin-producing strains of *V. cholerae *when the El Tor biotype is included, is one of the biomarkers for the pigment-producing strains, and may be of significance in biological research and epidemiological tracing.

We observed that the nontoxigenic O139 pigment-producing strains exhibited a rb4 ribotype. In our previous study, the rb4 isolates were cholera toxin gene-negative O139 strains, and this ribotype is clearly different from the other patterns of the toxigenic O139 strains that are cholera toxin gene positive [27]. All of the rb4 strains were isolated from patients, and an unknown pathogenic mechanism is presumed [27]. Though the O139 pigment-producing strains examined in this study were isolated from environmental water samples, their possible pathogenicity should not be excluded, particularly since such strains are isolated successively in some years. The study showed that the pigment-producing strain expressed more toxin-coregulated pilus and cholera toxin, by possibly mechanism which pigment production might cause induction of the ToxR regulon due to generation of hydrogen peroxide [23]. Strain 3182 is the toxigenic strain associated with the seventh pandemic, and it is speculated that this strain is more virulent than other strains on account of its pigment production, based on its role in *V. cholerae *virulence factor expression [23].

## 5. Conclusions

In summary, in this study we demonstrate that the pigment-producing *V. cholerae *isolates have mutations in the tyrosine metabolic pathway are highly clonal, and suggest that pigment production may confer a survival advantage to this clone in the environment. The possible contribution of pigment production to *V. cholerae *pathogenesis of those nontoxigenic O139 strains and toxigenic El Tor strain in humans is of considerable interest and worthy of further investigation.

## Abbreviations

FAH: fumarylacetoacetate hydrolase; HGO: homogentisate oxygenase; (*p*HPD): *p*-hydroxyphenylpyruvate dioxygenase; LB: Luria-Bertani; MAI: maleylacetoacetate isomerase; PFGE: Pulsed-field gel electrophoresis; *V. cholerae*: *Vibrio cholerae*.

## Authors' contributions

RW carried out the main part of experiments in this study and drafted the manuscript, WH participated in designation and discussion in preparing the manuscript, ZH, WY and YJ participated in Mutation frequency analysis, DB participated in PFGE, and BK revised the manuscript. All authors read and approved the final manuscript.
